# Supplementary material for: Continuous flow vortex fluidic-mediated exfoliation and fragmentation of two-dimensional MXene
Source: R Soc Open Sci. 2020 May 13;7(5):192255. doi: 10.1098/rsos.192255 (PMC7277261; doi:10.1098/rsos.192255)
Supplement: Supplementary Information [file RSOS192255supp1.pdf]

# Continuous flow vortex fluidic mediated exfoliation and fragmentation of 2D MXene

**Ahmed Hussein Mohammed Al-antaki[a,b], Suela Kellici[c] , Nicholas P. Power[d], Warren D. Lawrance[e], Colin L. Raston[a*]**

a Flinders Institute for Nanoscale Science and Technology, College of Science and Engineering, Flinders University, Adelaide, SA 5042, Australia.
b Department of Chemistry, Faculty of Sciences, University of Kufa, Kufa, Najaf, Iraq.
c School of Engineering, London South Bank University, 103 Borough Road London, SE1 0AA (UK).
d School of Life, Health & Chemical Sciences, The Open University, Walton Hall, Milton Keynes, MK7 6AA (UK).
e College of Science and Engineering, Flinders University, Adelaide, SA 5042.

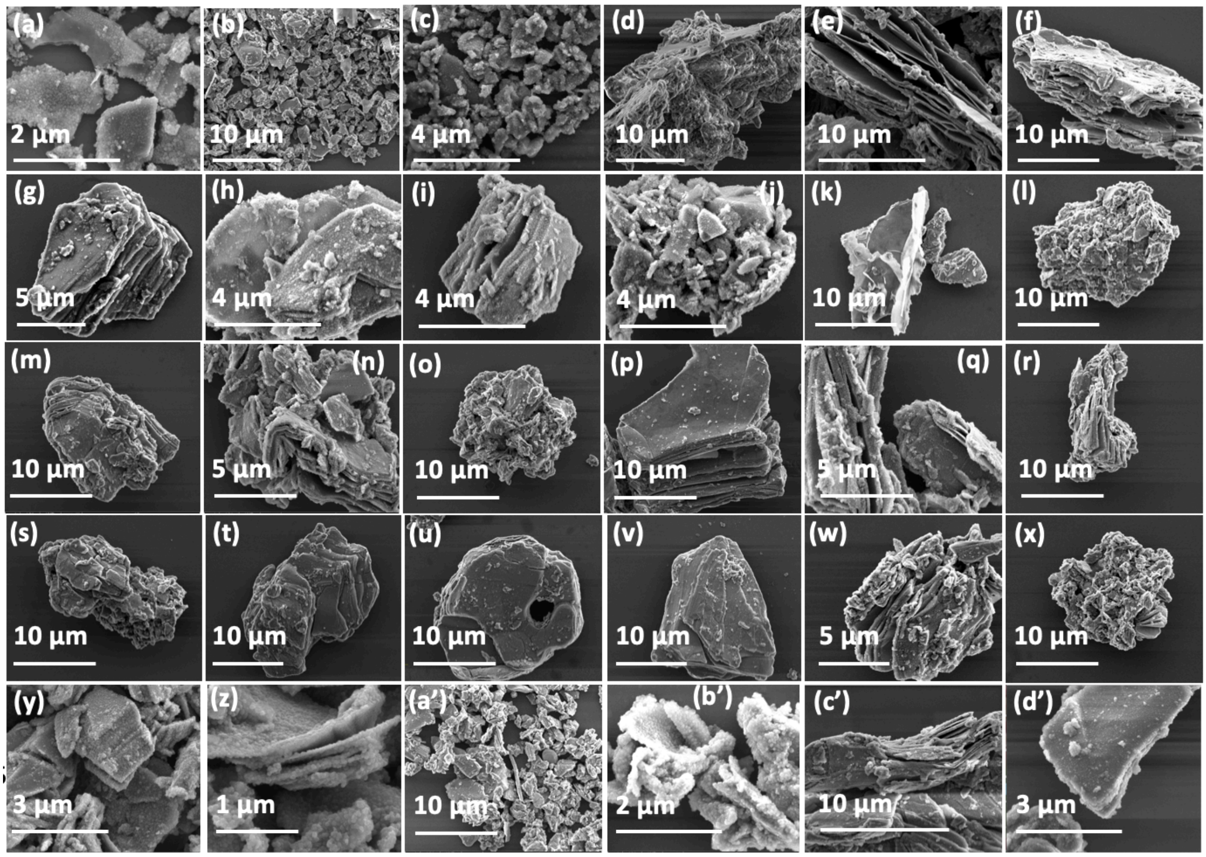

**Fig. S 1.** SEM images for MXene drop cast on silicon wafer, post VFD processing under air, θ 45° for 15 mins, concentration 1 mg/mL in. (a, b) DMF, rotational speed 4k rpm. (c, d) DMF, rotational speed 6k rpm. (e, f) DMF, rotational speed 7.5k rpm. (g, h) Toluene, rotational speed 4k rpm. (I, j) Toluene, rotational speed 6k rpm. (k, l) Toluene, rotational speed 7.5krpm. (m, n) *m*-Xylene, rotational speed 4k rpm. (o, p) *m*-Xylene, rotational speed 6k rpm. (q, r) *m*-Xylene, rotational speed 7500 rpm. (s, t) *o*-Xylene, rotational speed 4k rpm. (u, v) *o*-Xylene, rotational speed 6k rpm. (w, x) *o*-Xylene, rotation speed 7.5k rpm. (y, z) DMF and *o*-Xylene (1:1), rotational speed 4k rpm. (a’, b’) DMF and o-xylene as solvent (1:1), rotational speed 6k rpm. (c’, d’) DMF and *o*-xylene (1:1), rotational speed 7.5k rpm.

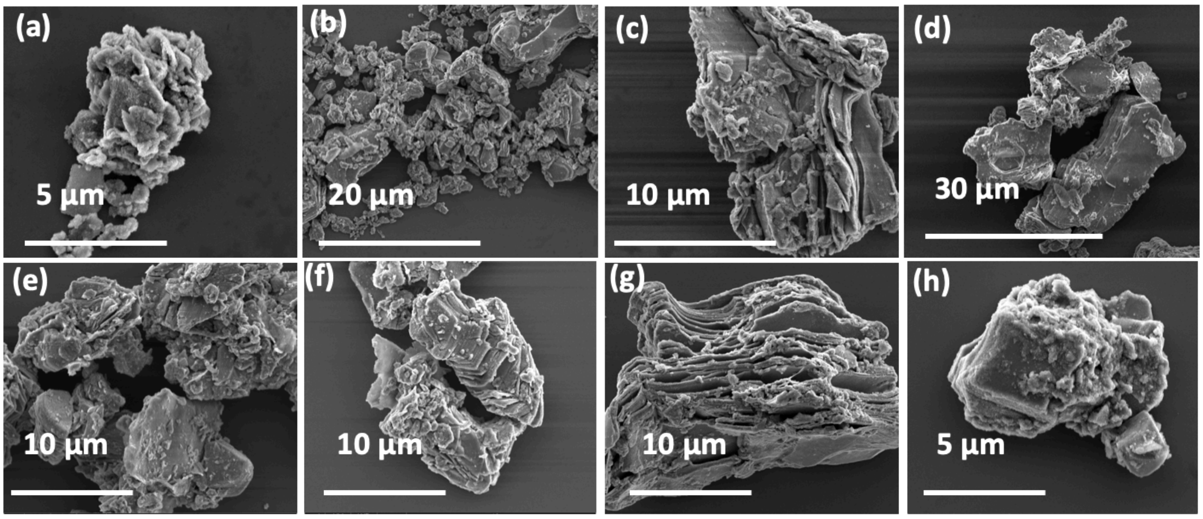


**Fig. S 2.** SEM images for MXene drop cast on silicon wafers, post VFD processing under air, θ 45° for 30 mins, rotational speed 4k rpm, concentration 0.5 mg/mL in. (a, b) DMF and IPA (1:1). (c, d) *m*-Xylene and IPA (1:1). (e, f) Toluene and IPA. (g, h) *o*-Xylene and IPA (1:1).

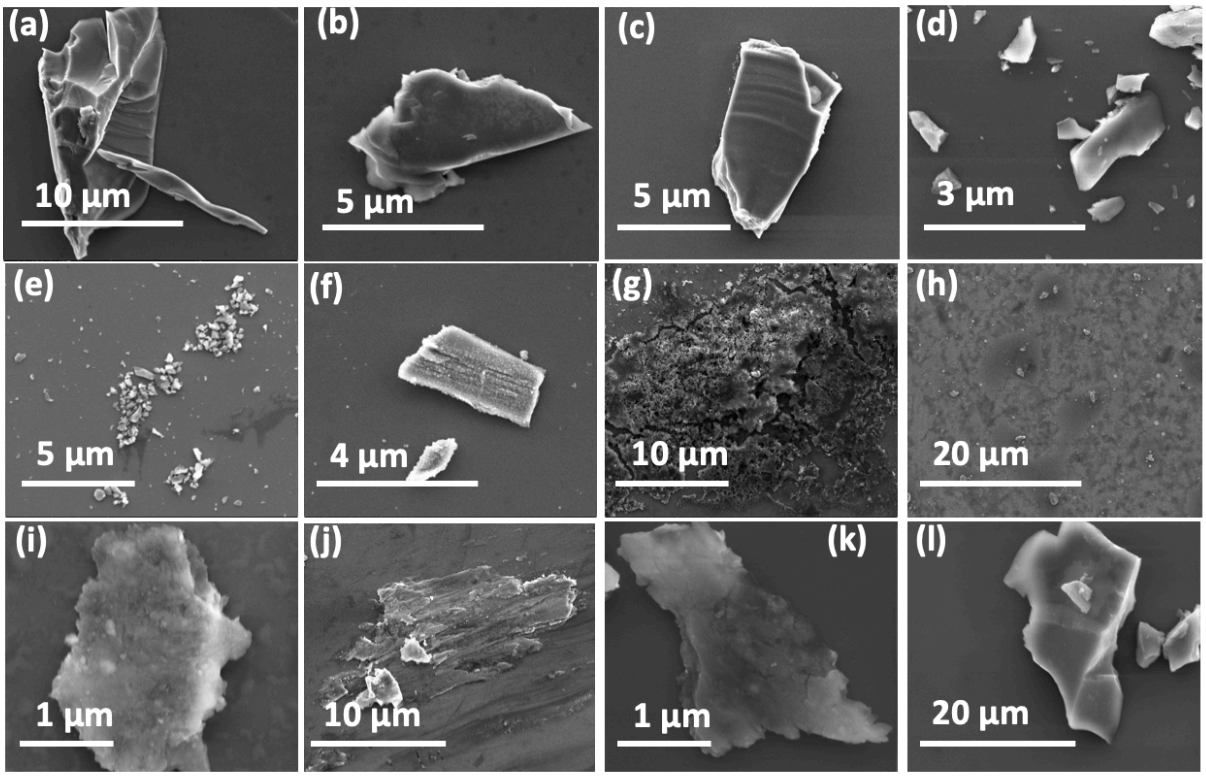


**Fig. S 3.** SEM images for MXene drop cast on silicon wafers, post VFD processing under air, θ 45°, irradiated quartz tube with a pulsed laser operating at 1064 nm, and concentration 0.5 mg/mL in IPA and water (1:1). (a, b) Rotational speed 4k rpm for10 min, laser at 260 mJ. (c, d) Rotational speed 4k rpm for 30 min, laser at 260 mJ. (e, f) Rotational speed 7.5k rpm for 30 min, laser at 260 mJ. (g, h) Rotational speed 7.5k rpm for 30 min, laser at 660 mJ. (I, j) Rotational speed 7.5k rpm for 30 min, laser at 460 mJ. (k, l) Rotational speed 7.5k rpm, flow rate 0.2 mL/min, and laser at 260 mJ.

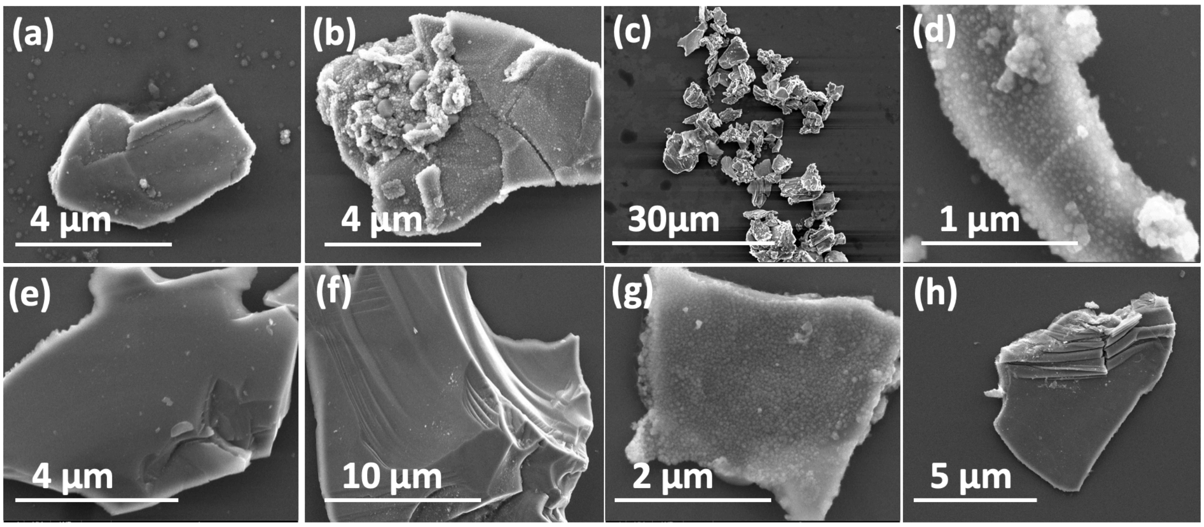


**Fig. S 4.** SEM images for MXene drop cast on a silicon wafer, post VFD processing under air, θ -45°, concentration 0.1 mg/mL in IPA and water (1:1), and flow rate 0.3 mL/min. (a, b) Rotational speed 2.5k rpm. (c, d) Rotational speed 4k rpm. (e, f) Rotational speed 6k rpm. (g, h) Rotational speed 8k rpm.

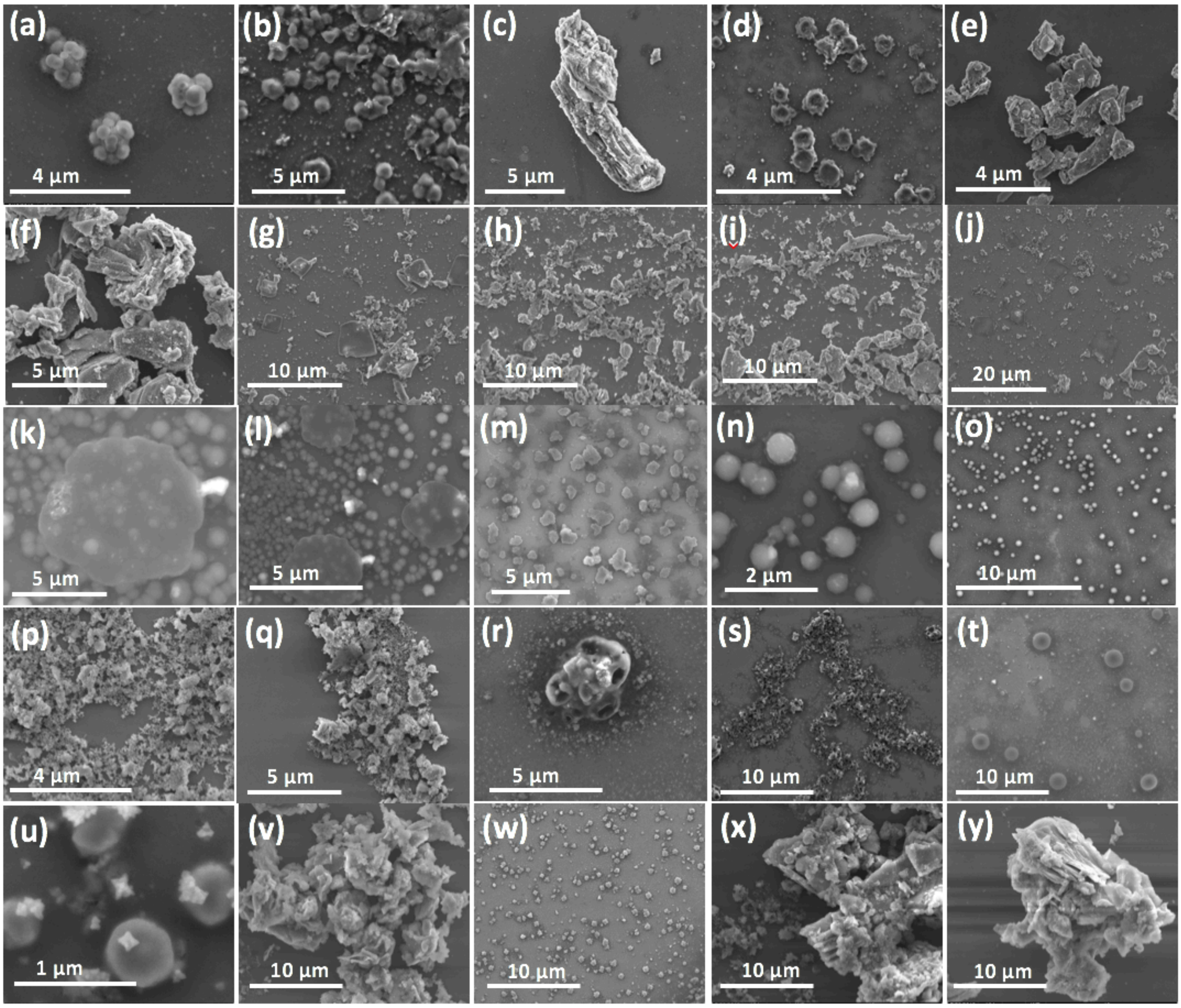


**Fig. S 5.** SEM images for MXene drop cast on silicon wafers, post VFD processing in water under $N_2$ gas, θ 45° and flow rate 0.5 mL/min. (a-e) Rotational speed 4k rpm, concentration 0.25 mg/mL. (f-j) Rotational speed 4k rpm, concentration 0.25 mg/mL recycled three times in the VFD. (k-o) Rotational speed was 4k rpm, concentration was 0.5 mg/mL. (p-t) Rotational speed 6000 rpm, concentration 0.5 mg/mL. (u-y) Rotation speed 8k rpm, concentration 0.5 mg/mL.

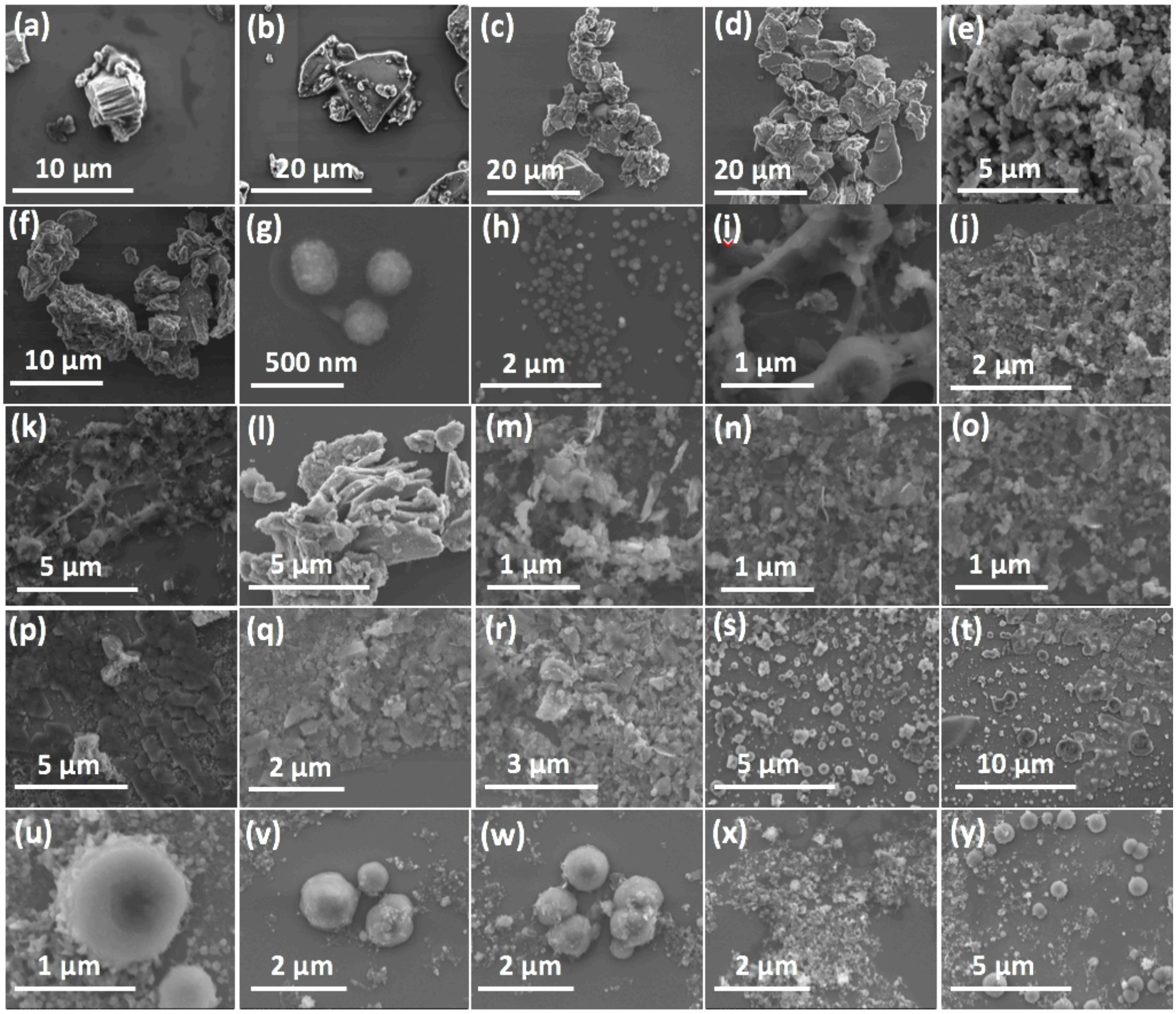


**Fig. S6.** SEM images for MXene drop cast on silicon wafers, post VFD processing under $N_2$ gas, θ 45°, (a, b) Concentration 0.25 mg/mL in DMF and *o*-xylene (1:1), flow rate 0.5 mL/min and rotation speed 4k rpm. (c, d) Concentration 0.25 mg/mL in DMF and *o*-xylene (ratio 1:1), flow rate 0.5 mL/min and rotational speed 4k rpm, recycled five times. (e, f) Concentration 0.25 mg/mL in ethanol, flow rate 0.5 mL/min and rotational speed 4k rpm. (g, h) Concentration 0.25 mg/mL in DMF and water (1:1), flow rate 0.5 mL/min and rotational speed 4k rpm. (i-l) Concentration 0.5 mg/mL in ethanol and water (1:1), flow rate 0.5 mL/min and rotational speed 4k rpm. (m-p) Concentration 0.5 mg/mL in ethanol and water (1:1), flow rate 0.75 mL/min and rotational speed 4k rpm. (q-t) Concentration 0.5 mg/mL in ethanol and water (1:1), flow rate 0.5 mL/min and rotational speed 5k rpm. (u-y) Concentration 0.5 mg/mL in DMF and water (1:1), flow rate 0.5 mL/min and rotational speed 4k rpm.

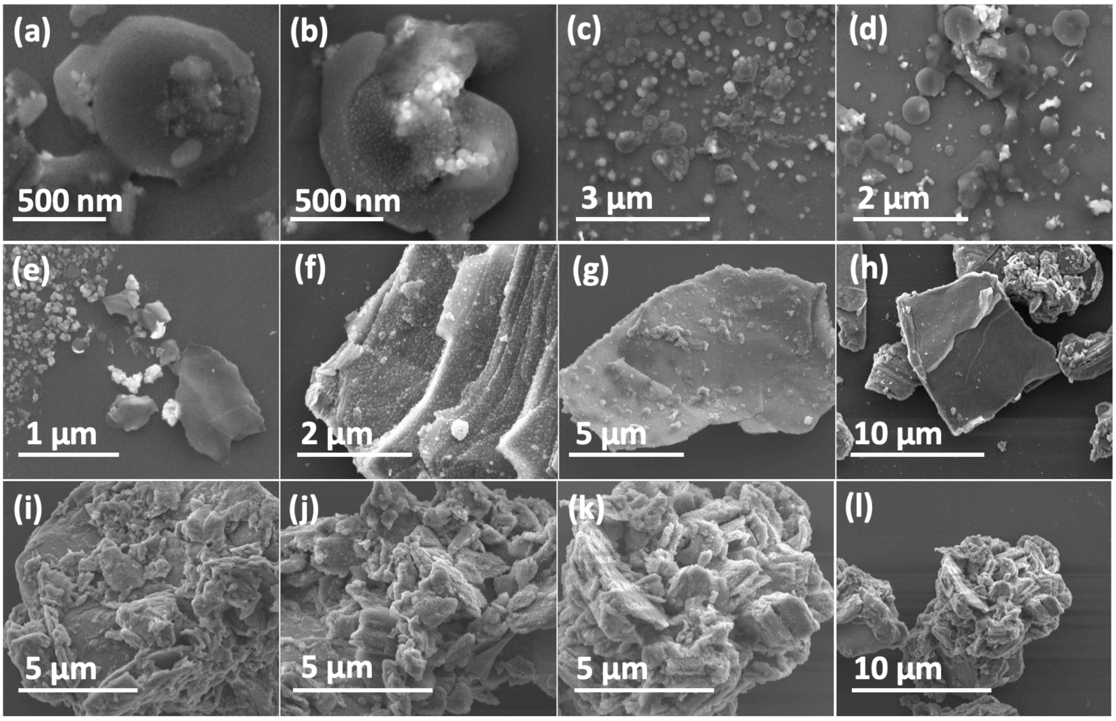


**Fig. S 7.** SEM images for MXene drop cast on silicon wafers, post VFD processing under $N_2$ gas, θ 45°, concentration 0.5 mg/mL in IPA and water (1:1) for 30 mins, the rotational speed 4k rpm to compare the result with a continuous flow.

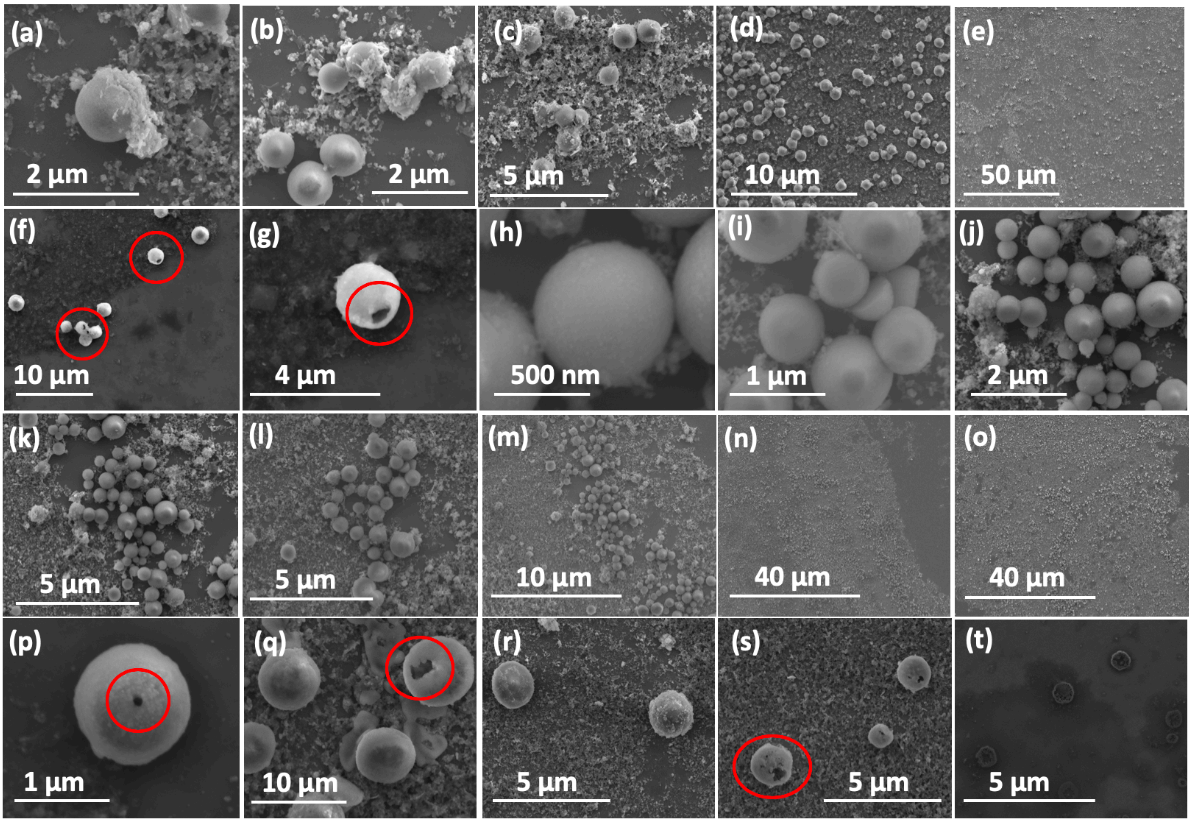


**Fig. S 8.** SEM images for MXene drop cast on silicon wafers, post VFD processing under $N_2$ gas, θ 45°, concentration 0.5 mg/mL in IPA and water (1:1) and flow rate 0.5 mL/min. (a-e) Rotational speed 4k rpm. (f, g) After three days for the same sample. (h-o) Rotational speed 5k rpm. (p-t) After one day for the same sample.

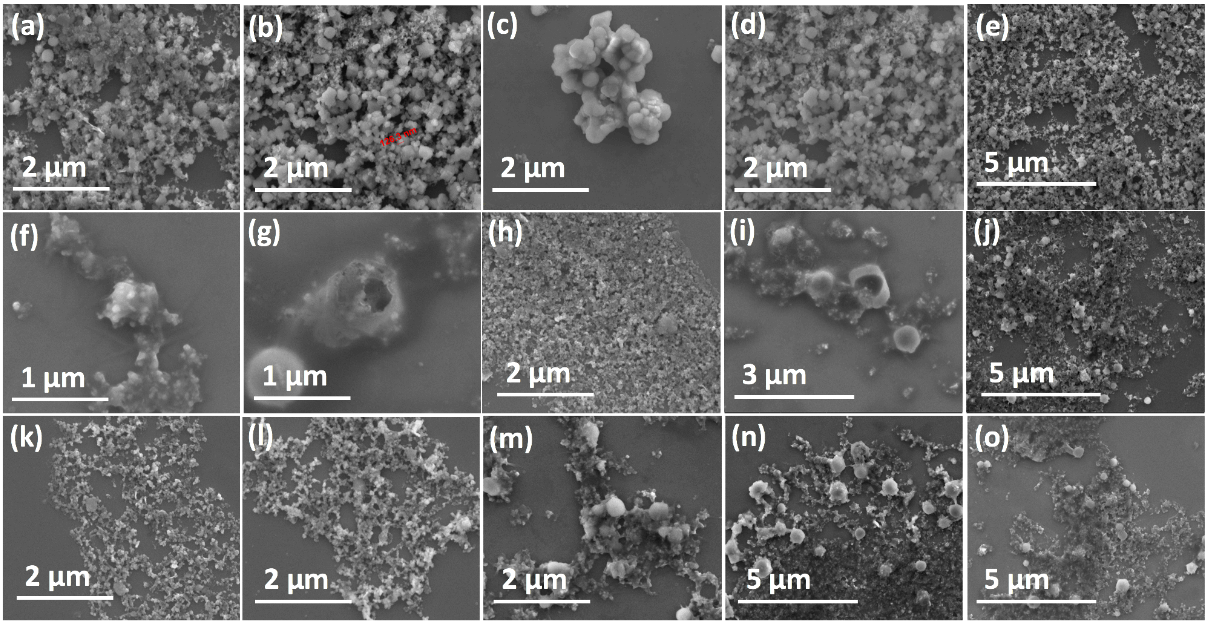


**Fig. S 9.** SEM images for MXene drop cast on silicon wafers, post VFD processing under $N_2$ gas, θ 45°, concentration 0.5 mg/mL in IPA and water (1:1) and flow rate 0.5 mL/min. (a-e) Rotational speed as 6k rpm. (f-j) Rotational speed 7k rpm. (k-o) Rotational speed 8k rpm.

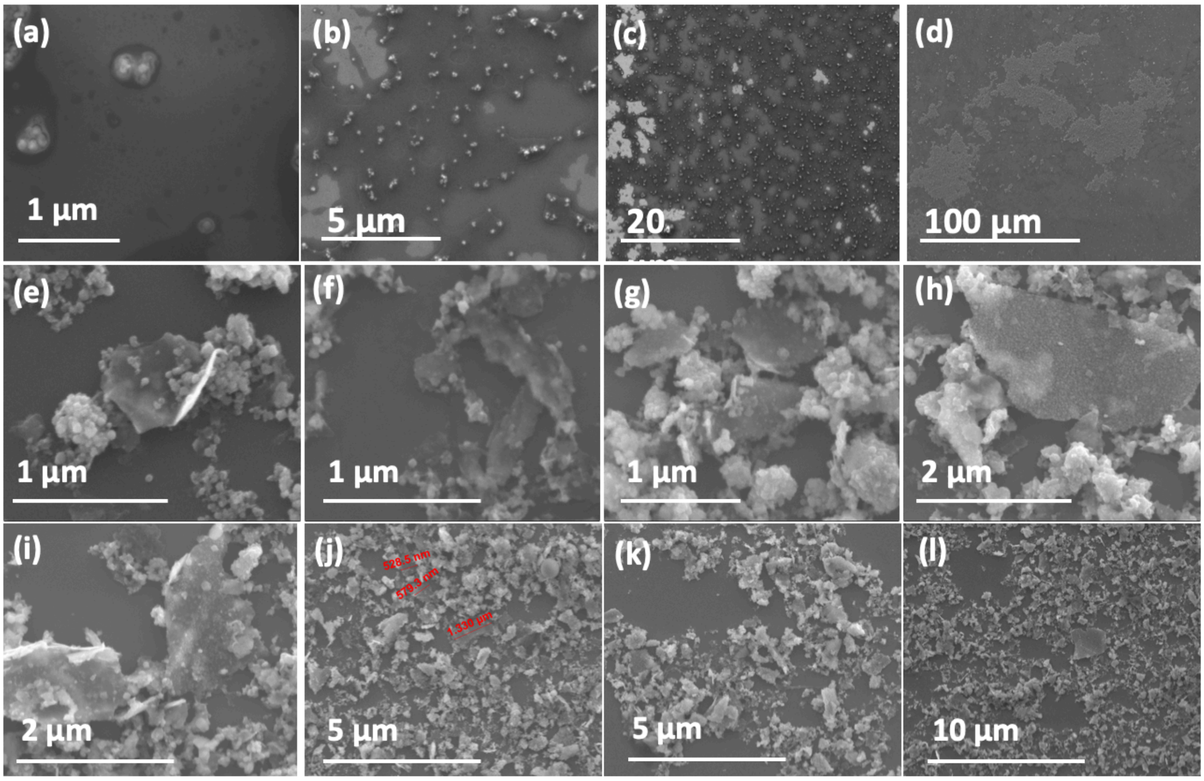


**Fig. S 10.** SEM images for MXene drop cast on a silicon wafers, post VFD processing under $N_2$ gas, θ 45°, concentration 0.5 mg/mL in IPA and water (1:1), rotational speed 4K rpm and flow rate 0.5 mL/min. (a-d) MXene nanoparticles (retained). (e-l) Exfoliated MXene sheets (retained).

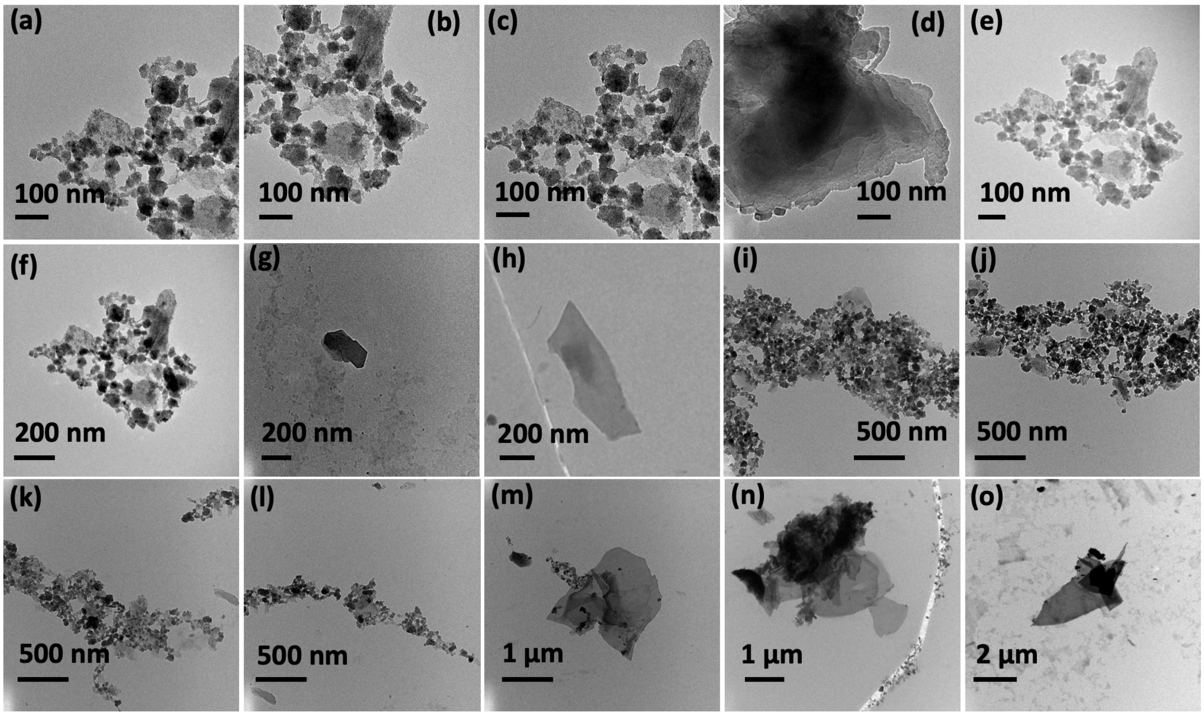


**Fig. S11.** TEM images for MXene drop cast on a grid, post VFD processing under $N_2$ gas, θ 45°, concentration 0.5 mg/mL in IPA and water (1:1), rotational speed 4k rpm and flow rate 0.5 mL/min.

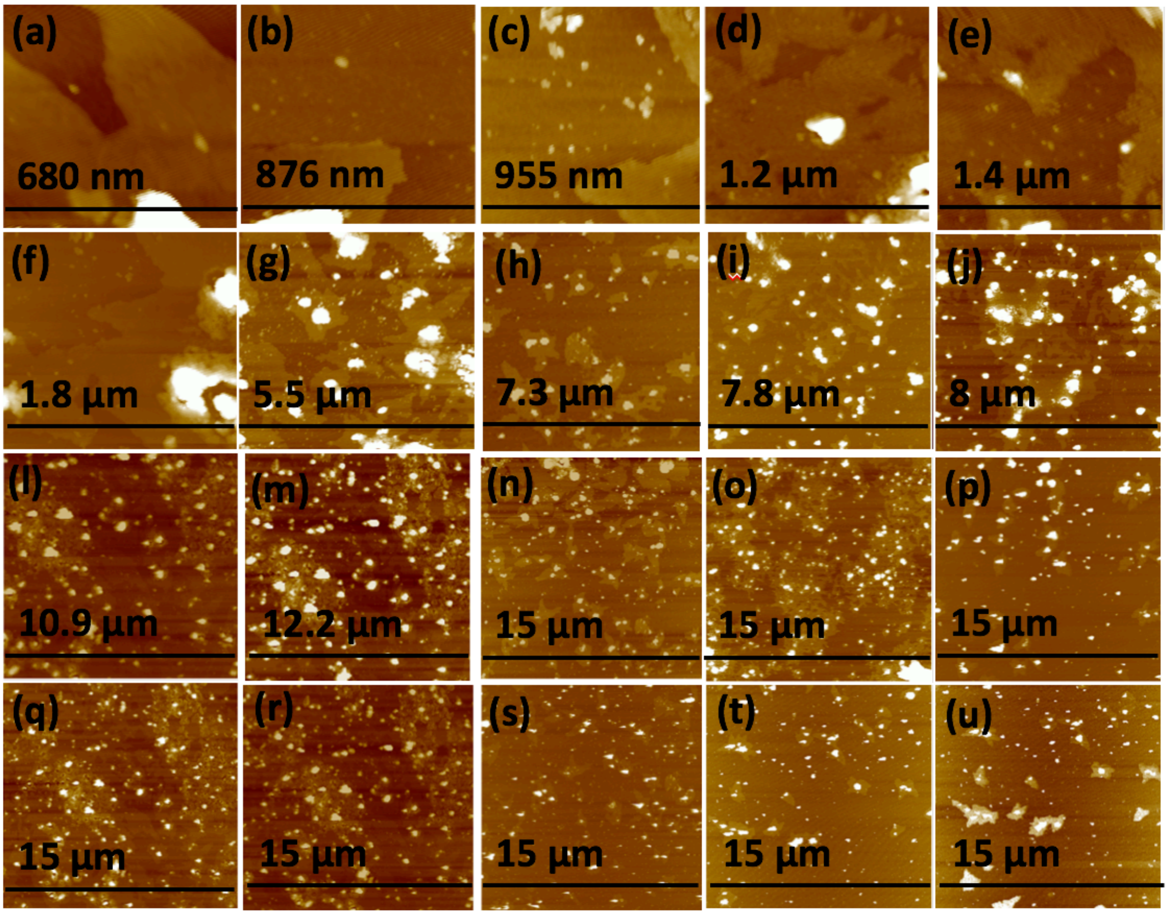


**Fig. S 12.** AFM images for MXene drop cast on a silicon wafers, post VFD processing under $N_2$ gas, θ 45°, concentration 0.5 mg/mL in IPA and water (1:1), rotational speed 4k rpm and flow rate 0.5 mL/min (collected and retained).

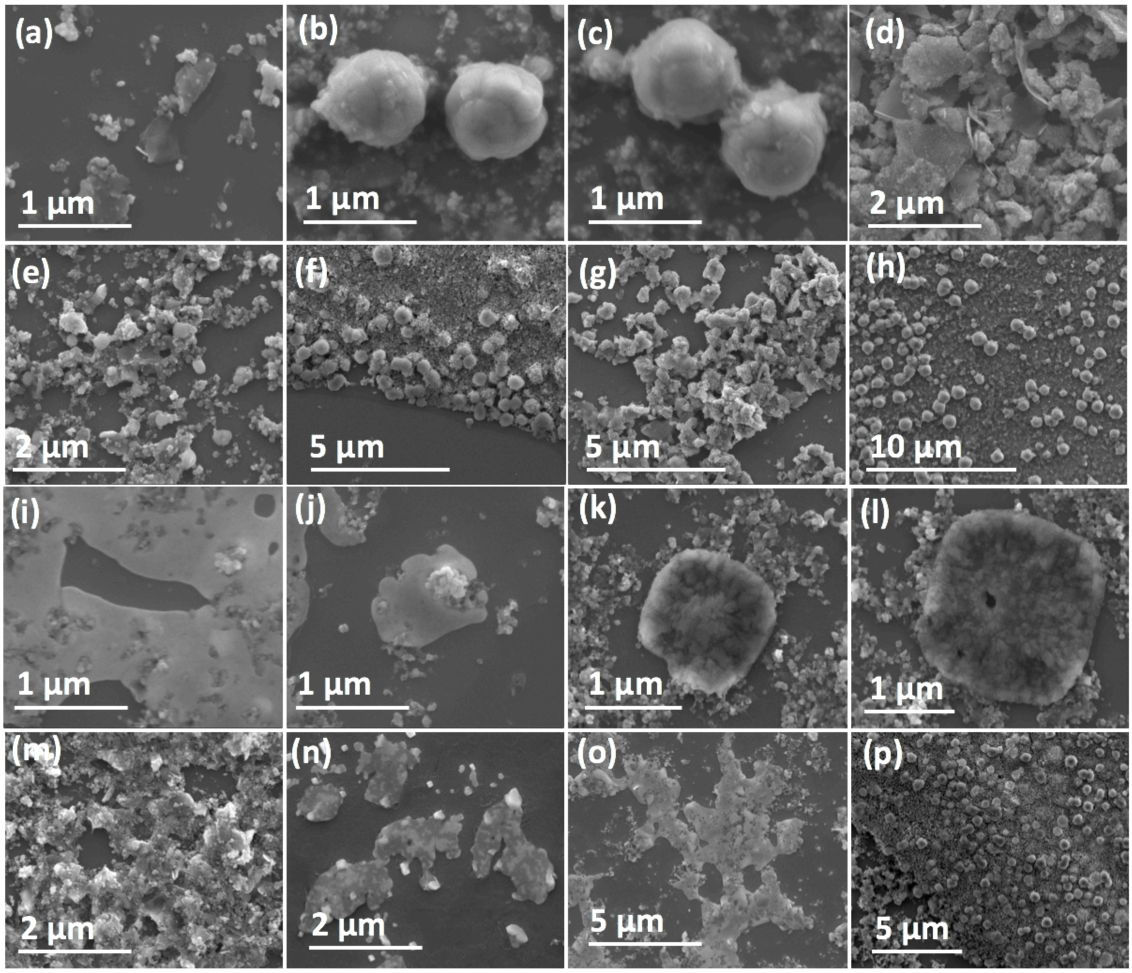


**Fig. S 13.** SEM images for MXene drop cast on silicon wafers, post VFD processing under $N_2$ gas, $\theta$ 45°, concentration was 1 mg/mL in IPA and water (1:1) and flow rate 0.5 mL/min. (a-h) Rotational speed 4k rpm. (i-p) Rotational speed 5k rpm.

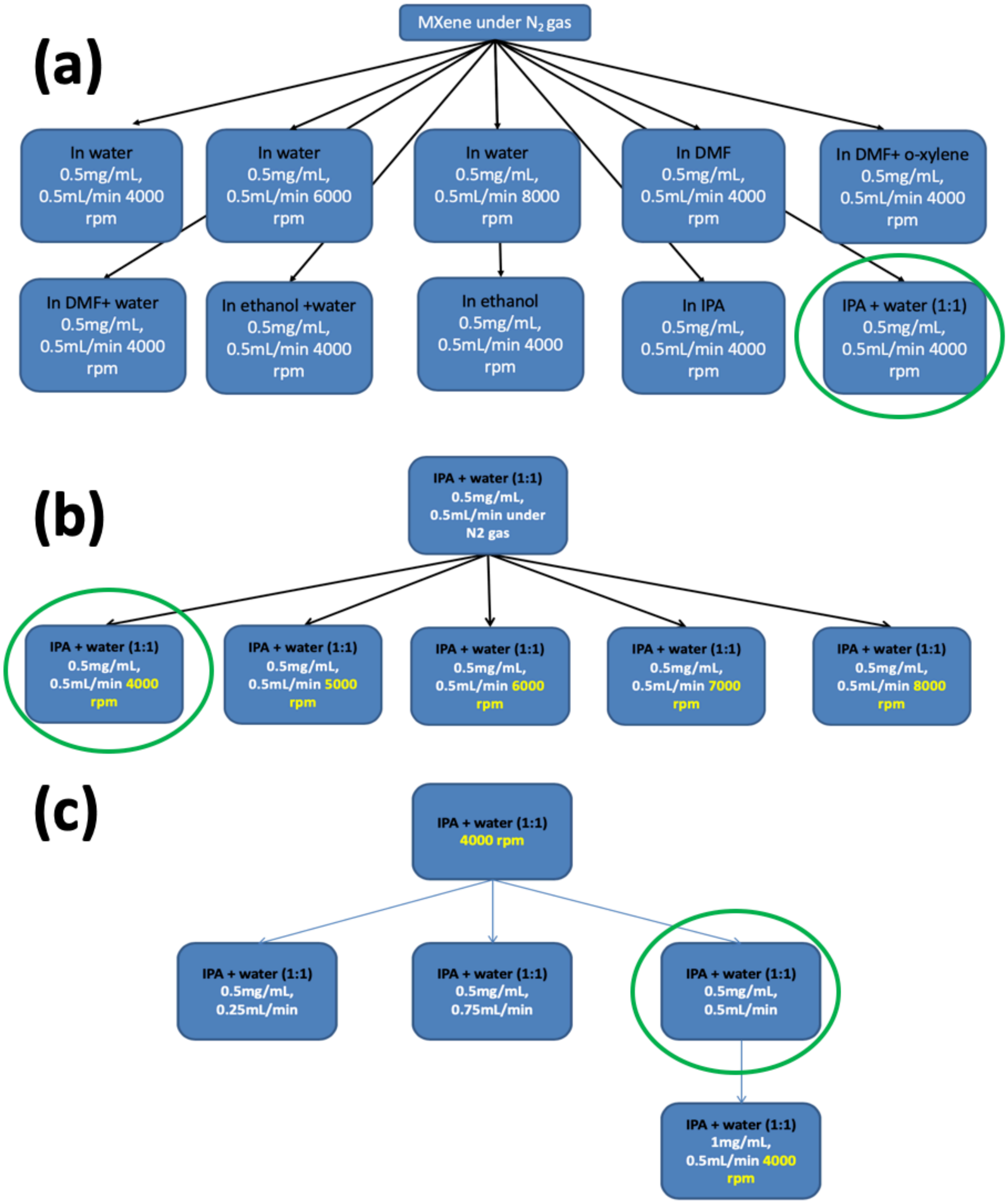


**Fig. S 14.** Schematic of the experiments used for processing MXene under $N_2$ gas. (a) Post VFD processing, θ 45°, concentration 0.5 mg/mL in different solvents for 0.5 mL/min flow rate, rotational speeds 4k rpm, and 6k rpm and 8k rpm for water. (b) Post VFD processing, θ 45°, concentration 0.5 mg/mL in IPA and water (1:1) for different rotation speeds and 0.5 mL/min flow rate. (c) Post VFD processing, θ 45°, concentration 0.5 mg/mL in IPA and water (1:1) for different flow rates of solution for 4k rpm rotational speed, as well as 1 mg/mL of MXene in IPA and water (1:1), θ 45°, and 0.5 mL/min flow rate using 4k rpm rotational speed.

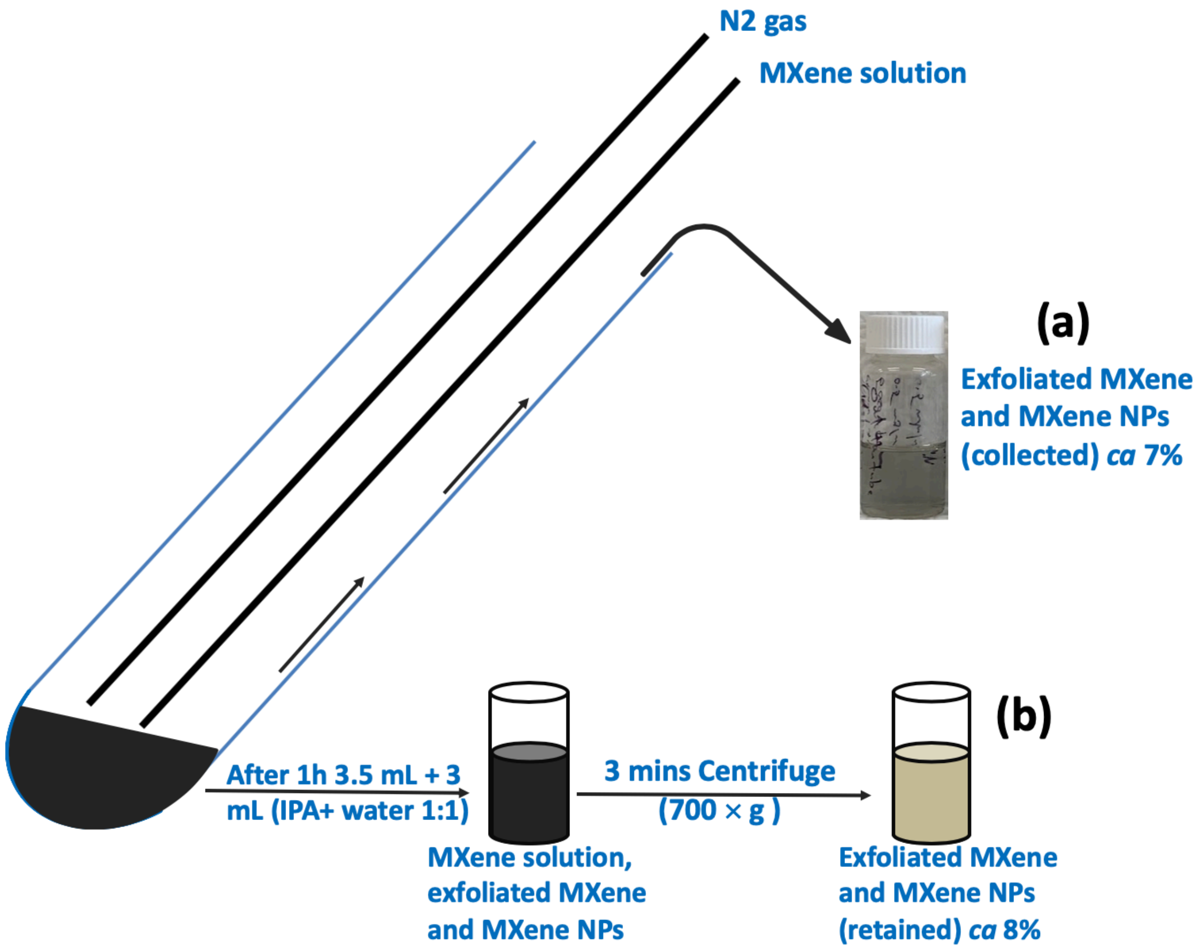


**Fig. S 15.** Schematic for processing MXene under $N_2$ gas, θ 45°, concentration 0.5 mg/mL in IPA and water (1:1), rotational speed 4K rpm and 0.5 mL/min flow rate. (a) Exfoliated MXene and MXene nanoparticles (collected). (b) Exfoliated MXene and MXene nanoparticles (retained).
